# Hepatitis C virus infection among patients with diabetes mellitus in Dammam, Saudi Arabia

**DOI:** 10.1186/s12913-016-1578-0

**Published:** 2016-07-27

**Authors:** Ebtesam M. Ba-Essa, Eman I. Mobarak, Nasser M. Al-Daghri

**Affiliations:** 1 Dammam Medical Complex, Dammam, Saudi Arabia; 2 Community Medicine Department, Faculty of Medicine, Alexandria University, Alexandria, Egypt; 3 Prince Mutaib Chair for Biomarkers of Osteoporosis, Biochemistry Department, College of Science, King Saud University, Riyadh, 11451 Saudi Arabia; 4 Biomarkers Research Program, Biochemistry Department, College of Science, King Saud University, Riyadh, 11451 Saudi Arabia

**Keywords:** Hepatitis C, Risk factors, Insulin resistance, Diabetes, Dammam

## Abstract

**Background:**

Data regarding comorbidities of hepatitis C virus infection (HCV) in the kingdom of Saudi Arabia (KSA) are lacking. The present study aimed to determine the prevalence and risk factors of HCV among Saudi patients with diabetes mellitus (DM) in Dammam, KSA.

**Methods:**

In this cross-sectional study done in 2011, a total of 1054 Saudi DM patients were randomly selected from the Diabetes Center in Dammam Medical Complex, KSA, for interview and HCV screening using the HCV Rapid Test. Positive cases were later confirmed via INNO-LIA HCV score line immune assay.

**Results:**

Seropositivity of HCV was 1.9 %. DM duration of >5 years increased the probability of HCV risk to 3.7 fold while insulin users were 3.2 times more likely to have HCV infection. Increased hospital admission (3–4 times) also increased HCV risk by 11.5 times and 13.6 times among patients with ≥5 admissions. Similarly, having 3–4 surgical procedures increased HCV risk by 8.6 times and 39.3 times with ≥5 procedures. HCV transmission is 4 times more likely by blood transfusion. Those who shared personal items were 8.5 times more likely to have HCV. Tattooing increased HCV risk by 6.7 times. The likelihood of HCV infection was also higher among DM patients with liver diseases and elevated liver enzymes.

**Conclusion:**

The study confirmed a significant association between HCV risk and DM. Frequency of HCV among DM Saudis was 1.9 %. Predictors of HCV among DM patients were sharing personal items, occupational exposure to blood or its products, elevated transaminases, tattooing, disease duration > 5 years, increased hospital admission and blood transfusion.

## Background

Diabetes mellitus (DM) is a global public health threat. In 2014, more than 387 million cases reported worldwide and is expected to rise by as much as 592 million by 2035 [1].

Hepatitis C virus (HCV) infection is another worldwide major health problem affecting more than 170 million, with 80 % harboring chronic infection. It is a major cause of morbidity, mortality and is a main risk factor for acute hepatitis and chronic liver diseases including cirrhosis, liver cancer, and chronic kidney disease [2].

There are increasing reports indicating an association between HCV and type 2 DM (T2DM). In controlled studies, individuals with HCV were observed to have increased T2DM risk [3, 4] and T2DM patients have at least a 2-fold greater risk of HCV infection [3, 5]. HCV induces impaired insulin signaling, insulin resistance (IR), disturbed glucose, protein and lipid metabolism as well as hepatic steatosis [6, 7]. It has been suggested that the associations between DM, HCV, IR and steatosis cumulatively increases risk of progression to hepatic fibrosis and hepatocellular carcinoma as well as reduce virological response to interferon-α-based therapy [7].

In the Kingdom of Saudi Arabia (KSA), DM is as a major public health problem with alarming rates [8]. Data regarding co-morbidity with HCV are lacking. The study therefore aims to determine the frequency and risk factors of HCV among Saudi patients with DM.

## Methods

The present cross-sectional study was undertaken last 2011. Target population was all known DM patients attending the Diabetes Center in the Dammam Medical Complex, Dammam, KSA. A random sample of consenting Saudi patients were selected every other day to be included in the study. Response rate was 100 %. Enrolled patients were interviewed in privacy by the investigators using a generalized questionnaire that includes socio-demographic characteristics, intravenous drug use, family history for DM and other medical histories. History of previous exposure to possible risk factors for HCV infection (at least 6 months prior to interview) were also collected. Weight and height were measured and noted.

A drop of whole blood per patient was collected by the investigators from the blood samples drawn for routine lab investigations. Following the manufacturer’s instructions, blood was tested for HCV antibodies (qualitative detection) using “The Hepatitis C Virus Rapid Test Device” (whole blood/serum/plasma) [ACON Laboratories, Inc. San Diego, CL, USA]. This test has an accuracy of 99.3 %, sensitivity of 99 % and specificity of 98.6 %. Patients with positive results were referred to the hospital central lab for confirmation via INNO-LIA HCV score line immune assay [Innogenetics, Belgium]. Confirmed HCV patients were referred to Hepatology department for management.

Patient records were obtained after the laboratory results of the routine lab investigations performed at the date of the interview [serum levels of liver enzymes; alanine aminotransferase (ALT) and aspartate aminotransferase (AST) and creatinine] were released. Levels of these markers were judged based on the upper limit of the reference normal range according to the used kits. High lipid profile was defined as >200 mg/dl for cholesterol, >150 mg/dl for triglycerides, >130 mg/dl for low-density lipoprotein cholesterol (LDL-C) [9]. Cut off for low level high density lipoprotein cholesterol (HDL-C) was <50 mg/dl for females and 40 mg/dl for males [9]. Glycemic control was classified into uncontrolled if HbAc_1_ **≥** 7 % and controlled if HbAc_1_ < 7 % [10]. The available findings of abdominal ultrasonic examination were also recorded.

Body mass index (BMI) was calculated as the ratio of the weight (kg) to the height(m)^2^ then classified as underweight (<18.5 kg/m^2^), normal (18.5–< 25 kg/m^2^), overweight (25–< 30 kg/m^2^), grade I obese (30–< 35 kg/m^2^), grade II obese (35–< 40 kg/m^2^) and grade III obese (≥40 kg/m^2^) [11].

### Statistical analysis

Data were analyzed using SPSS version 16.0 (SPSS Inc. Chicago, IL, USA) and the Epi-info (CDC**,** Atlanta, GA) [Open Source Epidemiologic Statistics for Public Health, Version 2.3.1. www.OpenEpi.com, updated 2011/23/06, accessed 2012/06/14]. The mean, standard deviation, median, odds ratio corresponding 95 % confidence interval (95 % CI) were computed. Chi-square and Student’s *t*-test were the used tests of significance. Logistic regression analysis was carried out using all measured variables to identify the relationship with other variables. The regression analysis was performed using the Enter method. A *P* value of <0.05 was considered statistically significant. Only significant predictors were presented with odd ratios and 95 % confidence intervals. Non-significant predictors with *p*-values >0.05 were not presented.

## Results

### General characteristics

The present study included 1057 Saudi DM patients (32.2 % males and 67.8 % females). The mean age of enrolled patients was 48.42 ± 13.70 year (min = 12, max = 87 years). At least 4.1 % of the subjects were minors (10–19 years) and 5.0 % were 20–29 years old. Adults between 30 and 39 years were 11.8 %. The largest proportion (79.1 %) of patients were 40 years old and above. Majority of the patients were married (91.8 %). More than 40 % were illiterate and 47.2 % attended schools. Only 5.0 % were university graduates or higher. Majority (74.9 %) were unemployed and were non-smokers (81.93 %). Overall, 89 % of DM patients were either overweight or obese (BMI ≥25 kg/m^2^; *n* = 1028).

### Disease profile

DM duration ranged from 1 month to 45 years (9.88 ± 7.84 years). T2DM was the most common (92.2 %). Patients were treated with insulin (27.3 %), oral hypoglycemic drugs (56.8 %), combination drugs (9.8 %), or just with diet control (6.1 %). Glycemic control was achieved by 64.3 % of patients whereas 35.7 % were uncontrolled. Most patients (76.1 %) had a positive family history of DM.

### History of exposure to risk factors for HC

Of the 969 married patients, 3.2 % had a partner with known HCV. Exposure to dental procedure was given by 57.7 % of the patients. Sharing personal items such as shaving devices/tooth brushes were reported by 2.3 % of patients. A small proportion received a type of injection. The majority of patients (81.9 %) were previously hospitalized at least once in their lifetime with a mean of 1.7 ± 1.4 (min = 1, max = 9) admissions. Two thirds of patients (66.2 %) reported 1 to 8 surgical procedures (1.4 ± 0.8). Half (50.0 %; *n* = 646) of married women had a history of abortion. Recipients of blood transfusion were 17.5 %. A minority (0.8 %) were on dialysis and 0.3 % (*n* = 3) had a previous diagnosis of hepatitis B infection. Liver diseases (other than HCV) were reported by 3.3 %. Only 1.3 % of patients were exposed to blood or its products in occupational settings. Tattooing was reported by 1.8 %.

Women were more likely to be exposed to HCV risk factors than men. Women were 4.5 times (Confidence Interval (CI) = 1.3–15.1) more likely to have a husband with HCV. They were also more exposed to dental procedures [Odds Ratio (OR) 10.9 (CI = 8.1–14.9)], hospitalization [OR 1.7 (CI = 1.3–2.4)] and surgical procedures [OR 2.0 (CI = 1.5–2.6)]. The probability was also higher among women with history of blood transfusion [OR 3.4 (CI = 2.2–5.3)], injections [OR 18.0 (CI = 4.4–73.9)] and intravenous drug use [OR 32.1 (CI = 4.4–232.4)]. On the other hand, men were 4.4 times (CI = 1.9–10.3) more likely to share personal items. No differences were observed regarding glycemic control, occupational exposure to blood or its products, tattooing, dialysis, history of hepatitis B or other liver diseases.

### Clinical laboratory and imaging findings

Laboratory results revealed both increased serum transaminases among 5.8 % and creatinine among 8.2 % of patients. Abnormal lipid profile was observed in 40.6 % patients with high serum cholesterol, 35.8 % with high triglycerides, 39.7 % with high LDL-cholesterol and 47.9 % with low HDL-cholesterol. Abnormal abdominal ultrasound was reported by 105 out of 210 participants that suggested fatty liver (49.5 %) and liver cirrhosis (0.5 %).

### Prevalence of HCV

Using the Rapid screening test, 22 (2.1 %) of the patients were found to be seropositive for HCV. On applying confirmatory test, only 20 cases were true positive resulting in a frequency of 1.9 % without significant difference between type 1 and type 2 DM.

### Risk factors for HCV

Patients who tested positive for HCV (51.6 ± 11.7 years) were older than those who tested negative (48.36 ± 13.73 years). Table 1 shows that DM duration >5 years increased the risk of’ HCV 3.7 folds (CI = 1.1–12.7). Insulin users were 3.2 times (CI = 1.3–8.1) more likely to have HCV than non-users. The frequency of hospitalization and surgical procedures were significantly associated with HCV risk. Those who were admitted to hospital 3–4 times were 11.5 times (CI = 1.4–94.4) more likely to have HCV and the risk increases to 13.6 (CI = 1.5–123.9) if admitted >5 times. Similarly, surgical procedures increased the risk for HCV by 8.6 folds (CI = 1.7–44.2) with 3–4 procedures and 39.3 folds (CI = 3.1–495.0) with ≥5 procedures than those who never had surgery. Blood transfusion quadruples (OR = 4.0, CI = 1.6–9.8) the probability of HCV infection. Those who shared personal items with others were 8.5 times (CI = 2.3–31.4) more likely to have HCV than those who didn’t. Tattooing also increased the risk of HCV to 6.7 folds (CI = 1.4–31.0). The likelihood of HCV is also higher among DM patients with other liver diseases (OR = 19.4; 95 % CI = 7.2–52.4) and elevated liver enzymes (OR = 12.3; 95 % CI = 4.8–31.5).

**Table 1:** Distribution of Patients and HCV risk

Factor	Positive to HCV		Negative to HCV		OR (95 % CI)	X^2^ (*P* value)
	No	%	No	%		
Sex:						
Male^a^	5	25.0	335	32.3	1.4 (0.5-4.0)	0.48 (0.49)
Female	15	75.0	702	67.7		
Marital status:						
Married	20	100.0	949	91.5	0.98 (0.97-0.99)	1.85 (0.25)^b^
Non married^a^	0	0.0	88	8.5		
Employment:						
Yes^a^	2	10.0	263	25.4	3.1 (0.7-13.3)	2.47 (0.088)^b^
No	18	90.0	774	74.6		
Type:						
Type I	3	15. 0	79	7.6	2.1 (0.6-7.5)	1.49 (0.20)^b^
Type II^a^	17	85.0	958	92.4		
Insulin Treatment:						
Yes	13	65.0	380	36.6	3.2 (1.3-8.1)	6.76 (0.009)*
No^a^	7	35.0	657	63.4		
Glycemic control status:					0.97 (0.4-2.5)	0.004 (0.950)
Controlled^a^	13	65.0	667	64.3		
Uncontrolled	7	35.0	370	35.7		
Duration of disease:						
>5 years	17	85.0	826	60.6	3.7 (1.1-12.7)	4.93 (0.019)^b*^
^≤^5 years^a^	3	15.0	409	39.4		
Hepatitis C partner:^c^( (*n* = 969)						
Yes	1	5.0	30	3.2	1.6 (0.2-12.4)	0.214 (0.482)^b^
No^a^	19	95.0	919	96.8		
Occupational exposure:						
Yes	1	5.0	13	1.3	4.2 (0.5-33.3)	2.11 (0.236)^b^
No^a^	19	95.0	1024	98.7		
Dental procedure:					0.9 (0.4-2.2)	0.061 (0.804)
Yes	11	55.0	599	57.76		
No^a^	9	45.0	438	42.24		
Injection:					0.7 (0.1-5.5)	0.096 (1.000)^b^
Yes	1	5.0	70	6.8		
No^a^	19	95.0	967	93.2		
IV Drug Use:					0.8 (0.1-6.3)	0.034 (1.000)^b^
Yes	1	5.0	62	6.0		
No^a^	19	95.0	975	94.0		
Hospitalization:						
Yes	19	95.0	847	81.7	4.3 (0.6-32.0)	2.352 (0.151)^b^
No^a^	1	5.00	190	18.3		
Number of admissions:						
0^a^	1	5.0	190	18.3		
1–2 times	8	40.0	675	65.1	2.3 (0.3-18.1)	0.614(0.692)
3–4 times	7	35.0	116	11.2	11.5 (1.4-94.4)	8.047 (0.007)*
5+ times	4	20.0	56	5.4	13.6 (1.5-123.9)	8.825 (0.012)*
Surgery:						
Yes	17	85.0	683	65.9	2.9 (0.9-10.1)	3.21 (0.094)^b^
No^a^	3	15.0	354	34.1		
Number of operations:						
0^a^	3	15.0	354	34.1	2.40 (0.7-8.5)	1.97(0.195)
1–2 times	13	65.0	639	61.6	8.6 (1.7-44.2)	9.50(0.020)*
3–4 times	3	15.0	41	4.0	39.3 (3.1-495.0)	21.07 (0.044)*
5+ times	1	5.0	3	0.3		
Abortion:^d^(*n*–646)						
Yes	9	69.2	314	49.6	2.3 (0.7-7.5)	1.96 (0.262)
No^a^	5	30.8	319	50.4		
Blood Transfusion:						
Yes	9	45.0	176	17.0	4.0 (1.6-9.8)	10.68 (0.001)*
No^a^	11	55.0	861	83.0		
Sharing personal items:						
Yes	3	15.0	21	2.0	8.5 (2.3-31.4)	14.89 (0.009)^b*^
No^a^	17	85.0	1016	98.0		
Tattoo:						
Yes	2	10.0	17	1.6		
No^a^	18	90.0	1020	98.4	6. 7 (1.4-31.0)	7.77 (0.048)^b*^
Liver disease:						
Yes	7	35.0	28	2.7	19.4 (7.2-52.4)	63.94 (<0.001)*
No^a^	13	65.0	1009	97.3		
Elevated liver enzymes: (*n* = 1015)						
Yes	8	40.0	51	5.1	12.3 (4.8-31.5)	43.55 (<0.001)*
No^a^	12	60.0	944	94.9		
Positive US finding:(*n* = 210)						
Yes	1	16.7	104	51.0	0.2 (0.02-1.7)	2.75 (0. 212)^b^
No ^a^	5	83.3	100	49.0		
Elevated creatinine: (*n* = 1024)						
Yes	3	15.8	81	8.1	2.1 (0.6-7.5)	1.48 (0.200)^b^
No^a^	16	84.2	924	91.9		
Dialysis:						
Yes	1	5.0	7	0.7	7.7 (0.9-66.1)	4.89 (0.142)^b^
No^a^	19	95.0	1030	99.3		
Total	20	100	1037	100		

^a^Reference category

^b^Exact test

^c^Calculated for married (*n* = 969)

^d^Each category was tested against reference category; Calculated for females (*n* = 646); *Statistically significant

Significant independent predictors of HCV among patients are shown in Table 2 (non-significant predictors were not presented). These include sharing articles (OR = 27.5, CI = 5.4–140.0), exposure to blood or its products in occupational setting (OR = 23.8, CI = 2.2–257.7), elevated liver enzymes (OR = 19.1, CI = 6.0–61.1), tattooing (OR=, CI = 7.7 (1.4–44.0), diseases duration >5 years (OR = 6.8, CI = 1.5–31.5), hospitalization >2times (OR = 4.7, CI = 1.6–13.6), and blood transfusion (OR = 3.67, CI = 1.2–11.0) This model classifies correctly 98.5 % of cases.

**Table 2:** Predictors of HCV

Predictor	Odds ratio	Adjusted odds ratio (95 % CI)	*P* value
Sharing articles	3.32	27.53 (5.42–140.00)	<0.001
Occupational exposure to blood	3.17	23.80 (2.20–257.72)	0.009
Elevated liver enzymes	2.95	19.13 (5.99–61.10)	<0.001
Tattooing	2.05	7.74 (1.36–43.96)	0.021
Disease duration > 5 years	1.92	6.82 (1.48–31.47)	0.014
Hospitalization >2 times	1.55	4.71 (1.63–13.61)	0.004
Blood transfusion	1.30	3.67 (1.23–10.95)	0.020

R^2^ = 0.055

Adjusted R^2^ = 0.323

*X* ^2^ (*p* value) = 57.133 (<0.001)

Model sensitivity = 98.5 %

## Discussion

There is evidence that DM patients are at higher risk of acquiring HCV infection and may be related to either the disease itself or frequent parenteral exposure [12]. However, some controlled studies failed to support this hypothesis [13]. The prevalence rate of HCV (1.9 %) in the present study is much lower than the 36 % reported from Pakistan [12] and 11 % reported from Nigeria [14]. It is also lower than the rates reported in France, UK and USA, which ranged from 3 % to 8 % [3, 15, 16]. However the rate revealed by the present study is comparable to the 1.3 % reported from Tunisia [14] and the 1.65 % reported in Greece [17].

The rate of HCV among blood donors in the central blood bank of Dammam was taken as control. The screening of 10,853 apparently healthy blood donors during the year of study identified only 8 confirmed cases of HCV (0.074 %) [Obtained from blood bank records through personal communication]. As for the whole Eastern province, the catchment area of Dammam Diabetes Center, the recorded rate of HCV among blood donors was 0.59 % (79 out of 13,443 during 2001) [18]. According to the Ministry of Health, 299 (0.011 %) cases of HCV have been reported in the same region (2,776,046 inhabitants) in 2011 [19]. Hence, the present study provides evidence of the higher likelihood of HCV infection among DM patients.

The reported HCV cases in KSA are low (0.0–0.02 %, in 2011) [19] and declining [18–20], probably due to routine screening of blood donors, low prevalence of intravenous addiction and wide practice of infection control procedures among medical personnel. Nevertheless, it should be noted that intravenous addiction is still a major factor for HCV transmission and as such, patients who confide such information should be further investigated to track and identify other users who are potential carriers of HCV. Socioeconomic development, mass education and increased awareness of the disease also played a role [20, 21] in keeping the prevalence of HCV at bay.

In the present study, sharing personal items that may be contaminated with infected blood and tattooing were defined as HCV predictors. The risk of HCV continues to be a great occupational threat [22]. In agreement, exposure in occupational setting among the studied patients was a predictor for HCV. Failure to adhere to universal precautions promote exposure to potentially infectious body fluids [22]. Consequently, blood transfusion was also identified as a predictor of HC [17, 23] as well as increased hospital admission [24]. In the present work, HCV risk was significantly associated with both hospitalization and frequency of surgical operations. Parallel findings were previously observed [23]. These suggest a nosocomial source of HCV infection in chronically admitted DM patients.

In agreement with prior investigators [4, 13] who observed increased HCV positivity with increasing age, patients positive for HCV were older than those who tested negative. The higher frequency in older age may be explained by more exposure to risk factors [5]. The studied women had a higher rate of seropositivity compared to men. Ndako et al. [14] recorded the same finding, while others observed a significant association of HCV with male sex [4, 5]. The present finding may be explained by the current significant female predominance regarding exposure to blood transfusion, hospitalization, surgical interference, parenteral injections and dental therapy which are known risk factors for HCV and may be related to their gynecological or obstetric history.

Consistent with Sotiropoulos et al. [17], the present study did not show an association between HCV and the type of DM, unlike the studies done by Jadoon et al. [5], Ndako et al. [8] and Ali et al. [12] who demonstrated a link between HCV and T2DM. Higher infection among insulin users may be linked to more frequent exposure to insulin injections and finger sticks for monitoring blood glucose [10] where unsafe injections or sharing contaminated equipment promote infection [2]. In contrast, Rudoni et al. [15] reported a negative association between HC, mode of treatment and use of finger stick devices in hospitalized patients.

Association between chronicity of DM and HCV in the studied patients may reflect increased exposure for HCV infection [8, 20]. Other studies didn’t support this finding [13, 17]. HCV had no association with glycemic control status in the present study whereas increased risk of HCV was associated with good control in some studies [5] and with poor control in others [13].

Chronic elevations of transaminases are common in both DM and HCV even without clinical manifestations in the liver [16, 25]. In the present study, elevated transaminases were a predictor for HCV infection, reflecting that HCV infection maybe responsible for liver function abnormality. Consistent findings were observed in other studies [16, 17]. Hence, screening for HCV among DM patients can be conducted using liver function tests.

The authors acknowledge some limitations. The predictors of HCV in the present cohort, while identified based on statistical significance had wide confidence intervals and therefore should be interpreted with caution. Nevertheless, these risk factors are in agreement with several other epidemiologic data and therefore still has enough clinical precision to make decisions on how best to proceed if such HCV risk factors are present in every patient. Further information may still be needed.

## Conclusion

The study confirmed a higher rate and a significant association between HCV and DM in Saudi patients in Dammam, KSA. The present study recommends screening for HCV among DM patients with elevated serum transaminases or those having DM for >5 years. HCV awareness and prevention campaign for all chronic DM patients are encouraged.

## Abbreviations

ALT, alanine transferase; AST, aspartate aminotransferase; BMI, body mass index; CI, confidence interval; DM, diabetes mellitus; HCV, hepatitis C virus; HDL, high density lipoprotein; KSA, Kingdom of Saudi Arabia; LDL, low density lipoprotein; OR, odds ratio; T2DM, type 2 diabetes mellitus
